# “I’m still here, but no one hears you”: a qualitative study of young women’s experiences of persistent distress post family-based treatment for adolescent anorexia nervosa

**DOI:** 10.1186/s40337-021-00496-4

**Published:** 2021-11-12

**Authors:** Janet Conti, Caroline Joyce, Simone Natoli, Kelsey Skeoch, Phillipa Hay

**Affiliations:** 1 School of Psychology and Translational Research Institute, Western Sydney University, Locked Bag 1797, Penrith, 2751 Australia; 2 School of Psychology, Western Sydney University, Penrith, Australia; 3 Chair of Mental Health, School of Medicine, Translational Health Research Institute, Western Sydney University, Penrith, Australia

**Keywords:** Adolescent anorexia nervosa, Maudsley family therapy, Family-based treatment, Experience, Identity, Qualitative

## Abstract

**Background:**

Family-based treatment (FBT) is the current treatment of choice for adolescent AN based on positive outcomes that include weight restoration in around two-thirds of adolescents. Nevertheless around a quarter drop-out from treatment, particularly in the earlier phases, and a notable proportion of treated adolescents are reported to experience ongoing psychological distress during and post-treatment. This study explores the under-researched experiences of these adolescents.

**Method:**

Fourteen participants from Australia, New Zealand and the United Kingdom were interviewed about their experiences of FBT. An inductive thematic analysis of interview transcript data generated key themes related to their experiences, identity negotiations and the discursive materials these used to construct these.

**Results:**

The participants identified working as a family unit as key to their recovery, highlighting the importance of family therapy interventions for adolescent AN. However, they perceived an almost exclusive focus on weight restoration in the first phase of FBT was associated with experiences that included a relative neglect of their psychological distress and a loss of voice. Key within these experiences were processes whereby the adolescent engaged in identity negotiation and (re)claiming of their voice and implicit in their family standing with them in the treatment was that their life was worth saving. What was noted as most helpful was when therapists advocated and took into consideration their unique needs and preferences and tailored treatment interventions to these.

**Conclusions:**

There is a need to develop and research treatments that address, from the outset of treatment, the adolescents’ psychological distress (including as experienced in the context of their weight restoration). This should be with priority accorded to the adolescent’s voice and identity negotiations, as they and their families take steps to address the physical crisis of AN and in doing so, support more holistic and durable recovery.

## Plain English summary

Family-Based Treatment (FBT) is a well-established, intensive approach to the treatment of adolescent anorexia nervosa (AN). The first phase of treatment focuses on eating and weight restoration, where parents are given responsibility for the adolescent’s home-based refeeding. This is followed by handing over of this responsibility to the adolescent with the final-phase a focusing on adolescent-specific psychological issues. While the majority of adolescents gain weight with this treatment, a substantive proportion experience ongoing psychological distress and around a quarter of families drop out in the first phase. Little is known about these adolescents’ experiences.

In this project, we interviewed 14 individuals who, with their family, had either dropped out of FBT and/or experienced ongoing psychological distress post-treatment. The participants noted that their parents’ stance in supporting them was life-saving and contributed to a sense that their life was worth saving. However, they also noted a relative absence of focus on their psychological distress, particularly in the early stages of treatment. Most helpful for participants was when therapists took into consideration and tailored treatments to them. Future treatments need to consider ways to support an adolescent’s psychological distress more comprehensively, prioritise their voice and support them in finding an identity outside of the AN identity.

## Background

Anorexia Nervosa (AN) is characterised by significantly low weight and an obsessional fear of gaining weight [1], frequent onset in the adolescent years [2] and mortality of 6–15%, with half of all deaths resulting from suicide [3–6]. A complexity for the treatment of AN is the egosyntonicity of the AN symptoms, many of which are valued by the experiencing person that contribute to a reluctance to engage in treatment [7]. For those who do engage in treatment, drop-out rates are high, including 23–73% in outpatient settings [8].

Family Based treatment (FBT) [9] has been reported to have positive treatment outcomes, particularly on eating disorder (ED) symptomatology, including in those adolescents assessed with higher ED symptomatology [10]. FBT has been proposed as the first line, ‘gold-standard’ evidence-based treatment for adolescent AN [11]. Through a number of randomized controlled trials (RCTs), FBT has been found to be associated with earlier weight restoration and reduced ED symptomatology and lower hospitalizations (and hence treatment cost), compared with adolescent focused individual therapy [12] and family therapy that addressed systemic concerns rather than eating related behaviours [13]. Further RCTs have found parent focused treatment (PFT) to be more efficacious than standard FBT; PFT being where treatment consists of the FBT therapist meeting with the parents separately to the adolescent who meets with a specialist nurse [14]. There is also evidence to indicate that when parental expressed emotion is higher, PFT results in higher rates of remission in the adolescent [15]. Early weight gain, a specific focus of FBT, has also been found to predict improved outcomes for the adolescent, particularly when there is a corresponding early focus on addressing negative emotion, parental criticism and the therapeutic alliance early in FBT and PFT treatments [16]. Furthermore, therapeutic alliance, as measured by an independent researcher-rater using the Working Alliance Inventory Observer Version (WAI-o) [17], has been found to be ‘achievable’ [18, 19] and ‘generally strong’ [20] in FBT. However, this measure of therapeutic alliance in these studies did not find a significant relationship and treatment outcome of remission of AN symptoms [18].

FBT is a three phase manualized treatment that begins with a focus on supporting parents to take responsibility for their child’s eating and weight restoration, progressing to phase 2 where this responsibility is re-allocated to the adolescent once they have increased their food intake and sustained reliable weight gain. The final phase commences when the adolescent maintains at least 95% of their ideal body weight, and is focused on re-establishing autonomy and developing a healthy identity [21, 22]; psychological processes and relational problems are not specifically addressed until this final phase of treatment [23, 24]. These phases of treatment are estimated to be delivered over 12 months over 20 sessions. Recent evidence in clinical settings has demonstrated that for those who do not meet remission by session 20, that further FBT sessions resulted in lower number of days in hospital and improved rates of remission compared to those who sought out alternative treatments to FBT [25].

A Cochrane review of 25 published and unpublished randomised controlled trials that compared family therapy interventions, including FBT, with other AN treatments [26] has found some evidence of a small effect size of FBT related to weight gain post-treatment, however, little evidence of differences between outcomes for groups across all comparisons of treatments, including in ED symptomatology (weight, ED psychopathology), drop out, relapse or family functioning measures at post-intervention or at follow-up. This review concluded that there currently exists insufficient evidence that family therapy interventions were superior to educational interventions and other types of psychological interventions for adolescent anorexia nervosa at long term follow up. There was also insufficient evidence of (1) outcomes outside symptom remission with only two trials of 21 reporting outcomes based on a return to normal functioning; and (2) whether one type of family therapy was more effective than others. Furthermore, the lack of detailed information about individuals and their families who drop out of adolescent AN treatments may have contributed to “the effect of artificially inflating the effectiveness of the interventions reviewed” [26].

Overall, results from RCTs show that FBT works well for less than half of the adolescents and their families who engage in this treatment [27]. Outside of this group for whom this intervention works well: (1) up to 27 percent of families drop out from FBT [28]; (2) 40% of adolescents continue with ongoing psychological distress despite weight restoration [29]; and (3) comorbid symptomology has a negative impact on treatment outcomes and increases rates of attrition [30–32]. In response to these findings, and given some promising findings for enhanced cognitive behavior therapy (CBT-E) adapted for adolescent AN [33, 34], CBT-E has recently been compared with FBT in a non-randomised effectiveness trial [35]. Findings indicated that greater early weight gain in FBT that by 6 to 12 months was indistinguishable between the two groups. Both treatments resulted in similar weight gains in general measures of psychopathology and clinical impairment; keeping in mind that the group who elected CBT-E were older and more unwell. Those who fared less well with these treatments reported a history of abuse and, specifically in the lower weight cohort, reported higher internalization, a presence of comorbid psychopathology, and prior mental health treatment.

A meta-synthesis of qualitative research into the experiences of family therapy for AN [36] has found three of 15 papers analysed adolescent and family experiences of FBT with one consisting of data generated from open-ended survey questions [37]. Two of these studies [37, 38] found that the most helpful aspect of FBT was parental support and understanding and the third study [39] found that the authoritative stance of therapists having an oversight of their eating behavior to be helpful. Less helpful was the neglect of issues other than AN [37, 39] and the adolescents’ unmet preferences for individual therapy [37]. There is increasing evidence to support the need for ED treatment interventions to focus on more rapid relief of the psychological dimensions of AN [40]. Furthermore, research into treatment experiences of those with a lived AN experience has identified a need for treatments to more directly address questions of identity, including the rebuilding a sense of identity outside of the ED identity [41, 42].

Overall there have been few studies that have focused on the experiences and identity struggles of those whose families either drop out of FBT and/or adolescents who continue with substantive psychological distress post-treatment [29]. This study sought to address this gap and give voice to those who experience persistent distress post FBT.

## The current study

The current study utilised a qualitative framework to give voice to the person with a lived adolescent AN experience with a focus on participant:

1. Experiences of Family-Based Treatment for adolescent AN in those who were interested in participating in research to improve the intervention; and
2. Identity negotiations in the context of this treatment intervention.

The aim of this study is to explore how these participant experiences and identity negotiations might inform future augmentations and transformative treatments for adolescent AN.

## Methods

### Design

This study was an inductive thematic analysis [43] with the understanding of themes as constructed within an interpersonal context. Analysis of data that comprised the themes/subthemes focused on some of the ways participants negotiated their identities through the discursive materials or language forms available to them at the time, the semantic and latent meanings they ascribed to the experiences of FBT and some of the dilemmas they faced [44] in the context of their recollection of experiences of FBT for adolescent AN. This study was also part of a larger study that explored the experiences and perspectives of parents who had experienced FBT [45] and clinicians who reported being trained and having practiced as FBT practitioners [46].

### Participants

A purposive sampling technique was utilised to invite participants to talk about their experiences of FBT and generate a context through which they could voice aspects of the treatment that was both helpful and their ideas of ways the intervention could be improved. These participants responded to advertisements via Australian clinicians; and after indicating their interest on completion of an eating disorders (ED) treatment experiences survey advertised through Facebook (see “Appendix A” for the wording of the research advertisement).

Fourteen participants aged 14–27 years (*M* = 18.58, *SD* = 3.20), who reported being diagnosed with AN and treated with FBT for adolescent AN on average 4 years earlier (range 1–14 years) were interviewed (in person or online/telephone). See Table 1 for further demographics and FBT treatment details. Half of the participants reported weight restoration post-treatment (with one later relapsing); all the participants reporting ongoing ED symptoms and psychological distress post-FBT. Eleven participants reported additional treatments for co-morbid psychological problems prior to or post FBT with three of these participants reporting psychological counselling whilst also engaging in FBT (see Table 2 for details).

**Table 1:** Demographic and treatment details

Participant Pseudonym	Family structure	Age at interview	Age at diagnosis	FBT age	FBT mths	Individual therapy + FBT	Phase completed	Weight restored after treatment
Abbey	Parents + 3C	27	13	13	12	Yes psychiatrist & psychologist	3	Yes
Amy	Parents + 4C	18	16	16	24	Psychologist-depression/ anxiety	3	No (recent AN hospitalization)
Beth	Parents + 2C	16	14	14	7–8	No	1 (D/C)	No
Charlotte	Parents + 3C	19	16	16/17	12	Psychiatrist	2 (D/C)	No
Harley	Parents + 3C	17	14	14	6	No	2 (D/C)	Yes (temporary) then lapsed
Hayley	Parents + 2C	14	10	11	24	CBT (at end FBT)	2 then lapsed (D/C)	No
Jessica	Parents separated + 3C	16	14	14	12	No	1 (D/C)	Yes
Kate	Parents separated + 3 C	18	17	17/18	3–4	No	1 (D/C)	No
Kaylee	Parents + C	20	16	16	6	No	1 (D/C)	Yes
Lydia	Parents + 3C	19	14/15	15	12	No	1 (D/C)	No
Maisy	Parents + 2C	19	14	14	24	CBT, mindfulness, Psychiatrist	3	Yes
Nora	Parents + 2C	21	14/15	15		No	1 (D/C)	No
Phoenix	Parents + 2C	18	15/16	15/16	7	No	3	Yes
Rachel	Parents + 2C	20	17	17	6–8	No	1 (D/C)	Yes

*C* children, *D/C* discontinued, *ED* eating disorder

**Table 2:** Additional treatments for Eating Disorder (ED) and other psychological problems

Participant Pseudonyms	Other eating disorder treatments	Eating disorder behaviours	Treatment for other problems
Abbey	Psychological therapy (including during FBT) Inpatient (P), BN day program	Restriction, over-exercise	Group Dialectical Behaviour Therapy
Amy	3 inpatient admissions (P), about to recommence hospital treatment, individual therapy(including during FBT)	Restriction, overexercise, Purging and binge eating	Psychiatrists/psychologists/school counsellor (P) for Depression, anxiety (included anti-depressants)
Beth	Nil	Restriction, over-exercise	Nil
Charlotte	Inpatient, psychiatrist	Restriction, purging	Nil
Harley	Inpatient then FBT (P), outpatient, multi-family therapy (P)	Restriction, overexercise, Purging	Psychiatrist/psychologist (C): Self-harm, Conversion Disorder, depression with psychosis: medication including SSRI’s, Seroquel (+ ”atypical antipsychotics”)
Hayley	Psychologist (Narrative Therapy) (C) Inpatient admission (P)	Restriction, overexercise	Psychologist (C): Anxiety
Jessica	Inpatient then FBT (P)	Restriction, over-exercise,	Saw Psychologist as a child (unsure why)
Kate	Psychologist (Narrative Therapy), dietitian, psychiatrist (C)	Restriction, overexercise	Psychologist and Psychiatrist (C): Anxiety; Family counselling before ED (P)
Kaylee	Inpatient then FBT (P) then inpatient	Restriction, over-exercise, Prior AN: purging, binge eating	Parent reported diagnosis ASD (“Asperger’s”), depression
Lydia	Inpatient then FBT (P) then inpatient (total 8 admissions), ED day program (C)	Restriction, over-exercise,	Nil
Maisy	Psychological therapy (including during FBT) + ED support group	Purging-reported once	Psychiatrist OCD—current treatment lithium, recently ceased escitalopram
Nora	Psychologist and psychiatrist (C) Inpatient admissions (P)	Restriction, overexercise, Purging	Psychologist and psychiatrists for depression, OCD (C)
Phoenix	Psychologists (P&C), paediatrician, dietitian, school counsellor	Restriction, overexercise Purging, binging	Depression, OCD
Rachel	Outpatient Psychologist and Dietitian, Group Therapy	Restriction	Counsellor for OCD (age 10 years), current anti-depressants

*C* current, *P* past, *OCD* obsessive compulsive disorder

### Procedure and materials

This study was approved by the Western Sydney University Human Research Ethics Committee (approval number: H11303).

Semi-structured interviews were carried out by two researchers (JC, SC) (see “Appendix B” for interview format) that were audio recorded, transcribed verbatim and de-identified with participants’ chosen pseudonyms. Transcripts were then given to participants to member check for accuracy and the removal of further identifying information for confidentiality.

### Analysis

The analysis was data-driven and inductive with all themes generated from the dataset [43]. Throughout the analytic process, themes were constructed through the explicit language used by participants, and an analysis of implicit meanings in their narratives, as language in this instance is assumed to construct a version of the participants’ experiences and meaning-making processes [47]. Three authors (KS, SN, JC) familiarised themselves with the data by reading and re-reading transcripts, coding meaningful units of raw data into draft themes and subthemes. Two authors (CJ, JC) also coded raw data into ‘nodes’ using QSR NVivo-12 qualitative data analysis software, examined the nodes for similarities and differences and grouped data related to the research question into categories. These categories of data were then collated and contrasted with the earlier drafted themes/sub-themes before being analysed for any further relationships and grouped together to generate overarching themes and a thematic map that addressed the research questions through in-depth analysis of exemplar data within each theme/sub-theme (JC, CJ, PH). Analysis by the researchers (see “Appendix C” for researcher positioning statements) traced some of the discursive materials these participants used to piece together key experiences and dilemmas associated with components of FBT and including patterns of identity negotiation [44, 48, 49].

The draft analysis was given to participants to member check for the purposes of validity (see “Appendix D” for member feedback provided by 4 participants) and to align analysis with participant feedback.

## Results

**Fig. 1:**
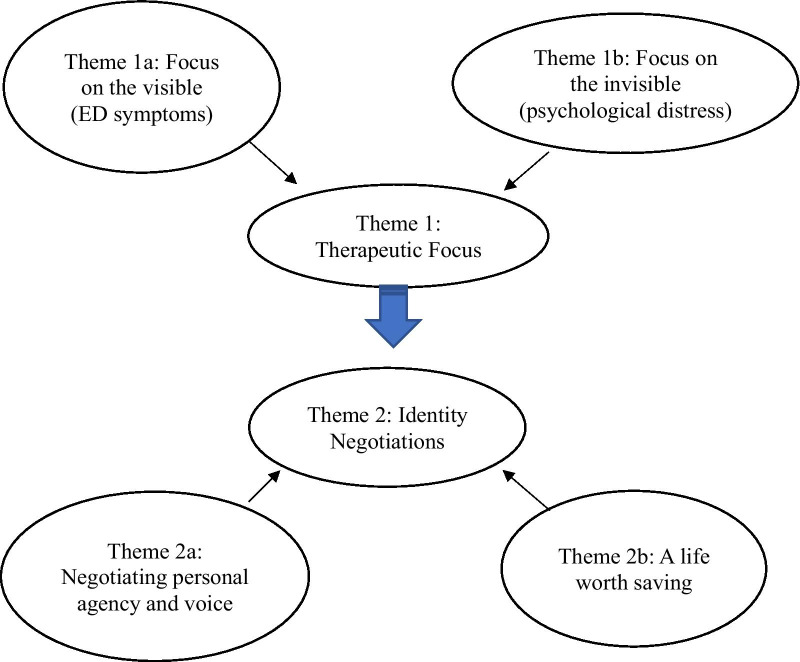
Thematic map: participants’ FBT experiences and identity negotiations

Analysis traced these participants’ experiences of key dimensions of the FBT intervention and ways they engaged in identity negotiations within these treatment contexts, including the reclaiming of identity and voice in matters related to their treatment (see thematic map, Fig. 1).

### Theme 1: Therapeutic focus

Participants recounted being both supported and distressed by the focus of Phase 1 of treatment on eating and weight restoration and handing control of these over to their parents. Of the 4 participants who reported completing the three phases of FBT, 3 concurrently had treatment with an individual therapist (Abbey, Amy & Maisy) and one participant retrospectively described being “taught” strategies by psychologists and dietitians during treatment to assist her with managing distress (Phoenix). Participant narratives highlighted that although FBT was preferred to inpatient treatment for most, when the therapeutic focus was predominantly on AN symptom reduction their emotional distress was obscured and further escalated.

### Subtheme 1(a): Focus on the visible

Ten participants’ narratives indicated that the early treatment focus of FBT, where their parents took responsibility for their eating, was experienced as a relief, albeit it was also a distressing experience.

*EXTRACTS 1*

*Pheonix:* *[…] As much as I hated it, and as much as I just wished for it to be over, it (FBT) did save me. The first few months were extremely distressing […] Knowing that I had no control whatsoever. [Later in interview] it was sort of down to business, let's do this as quick as possible to get your life back.*
*Kate:* *It was helpful…that saved my life. 100%. Going to that first appointment, they put me on eating plan. […] I’m glad that they got someone to be my eyes in that sense because I would not be here today.*
*Kaylee:* *The whole control aspect, it was, my parents had full control which made me feel real safe.*
*Abbey:* *[…] cause there was part of me that was, obviously I was fighting it but there was part of me that was like, “Oh thank god” like “I can’t get away with it. I just have to like sit and eat this”*

The allocation of responsibility for their eating to their parents was experienced as life-saving for these participants. The marking out of a boundary by which they were no longer responsible for their eating cultivated for some, a sense of relief and safety (e.g. Kaylee) and the possibility of getting “your life back” from AN, despite also being “extremely distressing” (Phoenix). Furthermore, the sense that others could “be my eyes” (Kate) indicated that these participants valued not only the support but also the alternative perspectives of others in assisting them to take steps to diminish the influence of AN on their lives. This included parents being supported by treatment teams to engage in nutritional nourishment despite a divided sense of self (“part of me … fighting it”; Abbey). Identified as helpful was when their parents were experienced as a steady support, informed yet understanding, cultivating safety whilst taking control over their eating that contributed to a containment of their distress in the context of the challenging time of nutritional restoration.

Alongside FBT being a life-saving treatment that addressed the medical crisis of AN, the participants also recounted parallel experiences of distress during and post FBT irrespective of extent of their eating and weight restoration and all of them acknowledged the significance of support by others for this distress, including family members.

### Theme 1(b): Focus on the invisible

Thirteen of the 14 participants argued that there was a lack of treatment focus on their experiences of psychological distress during FBT, including co-morbid psychological problems (see Table 2). The remaining participant (Maisy) reported FBT was experienced as “extremely pivotal to me getting better” and tailored to her preferences where her siblings attended only the first session. Individual therapy in addition to FBT was experienced for her as both an opportunity to “challenge my thoughts”, “learn how to self-soothe” through mindfulness techniques and a motivational intervention that worked “to convince me that recovery was a good thing”. She also highlighted the significance of seeing an individual therapist in addition to FBT who was “an outside person […] different from my parents”.

For ten of the participants who reported dropping out prematurely from FBT, an absence of focus on addressing emotional distress and interpersonal struggles was cited as a major contributor to this decision. For example;

*EXTRACTS 2*

*Harley:* *Because they never really addressed the underlying problems, it was all so much harder than it probably should have been, because I was still battling with the thoughts and battling with the guilt and all that.*
*Charlotte:* *My mum does have issues with her eating […] just made me feel like I was doing the wrong thing as a woman and as a female.[…] my sadness and how much I was hurting would also be expressed as anger[…] a lot of the times I would find it so difficult that I would ask to sit outside [of FBT sessions]. […] They[parents] hated seeing me so sad and I hated seeing them so sad, and it was just very confronting to have to bring that all up in family therapy and then not really take it further […] Like we opened old wounds and then they never really got closed and healed.*
*Beth:* *[…] […] I never really got a chance to properly, like talk out, like my anger, like with people. Like I never got to just express how I was really feeling, which is probably why I was so angry, because it was all, like building up inside, because I never got to express how I was feeling.*

The inadvertent effect of prioritizing of physical safety in phase 1 of FBT meant that these participants’ psychological safety was obscured with the implicit meaning taken from what was “going on inside” (Hayley) did not matter. For Charlotte, the structure of FBT meant the “old wounds” were opened and not healed, including the parallel process of eating difficulties of women in her family being left unaddressed contributing to an identity conflict where she was left questioning herself and to feelings of “sadness”. Implicit in these participants’ experiences of anger, was their valued stance and desire for openness within their family systems. They recollected their anger being responded to by being positioned as outsiders to their own therapy, losing their voice with systemic family issues left substantively unaddressed.

Within this context, a different sort of safety issue arose with six of the 14 adolescents reporting suicidal ideation during and/or post FBT treatment.

*EXTRACTS 3*

*Kate:* *[…] I never got to the point where I could really end my life but there were feelings of just wanted to end it because it’s easier than the voices that are in your head (crying, quite upset) and then you go into these sessions and they’re supposed to be sessions where you can let yourself speak your mind and you get time with the psychologist and it’s almost like, you just need to sit there and be quiet while everyone talks around you. They’re talking about you too (emotional).*
*Beth: * *I think I didn’t, like make it clear how, like depressed I was, and like the feelings of, like wanting to just end it all really. And I would never dare to say that in front of my mum, so I never did. And that’s, like kind of one of the things I was struggling with and struggled with it for a long time. And I thought I just never spoke up about it.*
*Harley (further quote from member checking):* *[…] Still to this day because of FBT if there’s a situation where I feel like there are links or similarities with control related or people talking at or about me like they did in FBT, my suicidal ideations are triggered. The focus on the impact of my family—which was a big aspect of FBT—as a result of the anorexia contributed to feelings of being a burden and secondarily, at times I wanted to kill myself because the FBT highlighted the damage I was doing to everyone around me.*

These participants argued that nutritional and weight restoration alone did not reduce their psychological distress; distress that continued largely unaddressed for them during the FBT intervention. Kate recollected experiences of a loss of voice where she was recruited into need(ing) “to sit there and be quiet”; Beth actively hid her distress, particularly from her mother; and Harley’s feedback on the analysis of her transcript (member checking) emphasized the lasting impression of treatment where being talked “at” or “about” continued to trigger “suicidal ideations” with continued identity associations of herself as a “burden” on others. These participants were active in their arguments that AN treatments need a greater focus on enabling the voicing, holding and processing of their and their family members’ emotional distress.

Parallel to the adolescent’s distress was family distress and conflict that was also experienced in the context of parents taking responsibility for their eating and weight restoration.

*EXTRACTS 4*

*Rachel:* *It [meals] just ended up being hard, plates smashed, tables turned, me punching, kicking and screaming, […] and then at the end of the time and it’d be like four hours later and we’d both be exhausted; no food would’ve been eaten.*
*Kaylee:* *[…] I didn't know who to listen to or who to follow (my parents or my urges) and so those were the destructive ways I dealt with them. For my parents, they wouldn't have known why I was acting out either, as I couldn't verbalise what was happening internally. So they responded to my outburst with screaming and anger and physical restraint towards me because I assume in their eyes, I was just being difficult about eating. Their reaction made the stress in my head even worse and made me even more suicidal, but I don't blame them for how they responded.*

Increased conflict in the family was evident in the participant narratives, with examples cited including angry outbursts with four participants disclosing anger that was physically expressed through parental restraint such as being held down or against a wall. Kaylee clarified that she was not blaming her parents for their responses at the time, that included physical restraint, however, she also recounted how these responses contributed to further distress, including suicidal ideation.

On the other hand, some of the participants talked about their experiences of FBT therapists advocating for them and mobilizing their parental support in ameliorating a sense of being “alone” in addressing AN.

*EXTRACTS 5*

*Amy:* *[…] in comparison to me going alone to therapy, my parents are actually getting an insight of where I’m at, […] the therapist, just to bounce off what I’ve said, from her conclusion, and what she feels is okay and what’s not okay, and um just I guess, in my best interests, what I should be doing, for her then to talk to my parents about it. Like that was—that was good.*
*Kaylee:* *[…] it definitely did teach my family them some things, and it definitely educated them on what would be helpful for me and what wouldn’t be helpful. And I think it was nice not to feel alone; it was nice to have my family there. So, it wasn’t just, like by myself.*
*Charlotte: * *[…] it taught them a lot about the tricks and the way that I was managing my eating disorder and keeping up with hiding food and water loading and making sure that I’d gained such and such amount of weight before the next appointment, […] and how it manifested itself in different ways, and different habits, and so then they were then able to become a lot more on top of it.*

These extracts exemplify key components of the therapeutic alliance that included FBT therapist advocacy, scaffolding insight into themselves and others’ understanding of their experiences (Amy) and ameliorating a sense of being alone in treatment through engagement with families in the intervention (Beth), including in standing together against AN (Charlotte).

### Theme 2: Identity negotiations

Alongside the participants’ treatment experiences were their parallel identity negotiations that were shaped by their experiences of personal agency and voice throughout treatment and their engagement in the FBT practice of externalisation of the illness (theme 2a). Furthermore, the implicit meanings ascribed to their parents’ support was that their life was worth living (theme 2b).

### Theme 2(a): Negotiating personal agency and voice

All the participants, at some point in their narratives, talked about struggles to negotiate personal agency and voice in their treatment, particularly in the first phase of FBT where their parents were allocated responsibility for their eating restoration.

*EXTRACTS 6*

*Phoenix:* *Because at stage one I definitely felt like a monkey in a cage and I had no control. My parents were doing everything for me.*
*Kate:* *[…] I felt tiny. I felt like everyone was overpowering to me and it was, I would just shut up and shut down. […] I just didn’t feel like a person. […] You feel like you’re getting treated like just someone who’s sick…it’s not the way you want to be seen.*

These extracts exemplify the identity negotiations of participants as they ascribed meaning to their parents being asked to take responsibility for their eating in the first phase of FBT. Phoenix’s use of the metaphor of a “monkey in a cage” depicted her experiences of a loss of personal agency, being monitored and unable to escape. The participants ascribed a number of negative identity conclusions in the context of their parents taking responsibility for their eating—for example for Kate this reinforced the identity of herself as a “sick” person that did not fit with who she understood herself to be.

Furthermore, parent’s taking responsibility for their eating had real effects on some of the participant’s relationships with their parents that had unintended impacts on their sense of themselves as a daughter.

*EXTRACTS 7*

*Lydia:* *[…] it [treatment] really fractured our relationship […] at that point, there was such a high level of conflict all the time, mutual distrust. […] , I think I lost my sense of that [self as person and daughter] and it’s almost as though I regressed and I was um, I was acting how I was being treated.*
*Harley (member checking):* *FBT ruined a previously strong relationship and caused my parents and siblings their own psychological unease and detriment. This contributed to a loss of myself and my identity and resulted in further destructive behaviours.*

A loss of voice was recounted by eight participants in the context of externalisation of the illness where they too experienced themselves as externalised with the AN.

*EXTRACTS 8*

*Nora:* *[…] just the fact that you had an eating disorder meant they were dismissive of anything you say, they believed anything you say was completely motivated by the eating disorder […] I was very distressed by that because I thought I’m still me, I’m still here, I can recognise that I have anxiety and unhelpful thoughts but I can still communicate as a person. […] I’m still me.*
*Lydia:* *I think to a certain degree, the treatment team had drilled into them [parents] that um I was not a person, I was an eating disorder and giving the reins to an eating disorder.*
*Charlotte:* *I was just infuriated that, you know, I’m trying to say something or have a conversation with my mum, and she’s referring to like anorexia and not Charlotte, telling Charlotte to come back whenever. Um, and I was like, “No, listen, like listen to me. I’m trying to tell you something”—that was very difficult—I’ve never really used that separation terminology until probably now, […] my eating disorder was me, […] That was my talent, that was what I was good at, that’s what I excelled in because I’d lost a lot of my identity, so I felt that that was my identity. So when—when people would refer to not say that they were talking to Charlotte I’d be like, “Are you kidding me?” But now I can see that that’s different and I can see the difference.*

These extracts exemplify the potential effects of the FBT practice of externalisation of the illness that led to the person’s voice being assumed by others to be the AN/ED. This misappropriation of the person’s sense of self (“I’m still me”; Nora) to the disorder by others (“I was an eating disorder”; Lydia) contributed further to a loss of voice and exacerbation of distress. Charlotte traced  the problem of externalization when built on the assumption that it is possible to achieve “separation” of the person from the ED when their identity is invested in the egosyntonic dimensions of the experience (“I felt that was my identity”). These extracts highlight the unintended consequences and struggles when the practice of externalization of the illness aimed to completely separate AN from the person and neglected to take into consideration the identity investments into the egosyntonicity of AN.

On the other hand, five of the participants found the process by which their therapists engaged them to externalize AN/ED to connect with a sense of identity outside the AN.

*EXTRACTS 9*

*Phoenix: * *[…] as I was restoring weight and as I was getting better and given more privileges and so on that I got to really find out who I was. […] they made us draw a Venn diagram with two circles. And they named one side “Phoenix” and one side anorexia and then throughout treatment they would make me draw where I thought the circles were overlapping and there was definitely a correlation between the distance of those circles and the amount of weight I restored. As I got healthier, the circles grew further apart and anorexia was separated from me*
*Rachel: * *They just called it, “the eating disorder.” […] And they’d be like, “What would your eating disorder say to this? Now sit in this chair and it’d be like, what would you say to this?” […] that was helpful, but they just didn’t do it enough. Like, it was just so much about food but they needed to care about my feelings.*
*Kate: * *[…] the other good thing about the FBT method was, they did really try and separate the person from the eating disorder. So you weren’t ever talking about, like, you could tell when someone was talking through the voice of the eating disorder or talking through their own voice. That’s what they tried to really distinguish.*
*Abbey:* *The anorexia. This is me genuinely saying something to you. Um, and also just for my own identity, um, and seeing the shift and the balance go. Like my identity increase and that decrease um, that was, that was really helpful. But mainly it was expressing my opinion to others that it was most, most helpful.*

Emerging across these participant narratives was their preference for a person-centred approach where the practice of externalization was focused on the person’s identity outside of the AN rather than primarily on elimination of the AN. Phoenix recounted a process of “finding out who I was” through the process of being given “privileges” as she gained weight and tracing her shifting relationship with AN as both individual and overlapping entities. Rachel was invited through a chair technique to have a dialogue with AN to enable her to reclaim her voice and preferences from AN; she argued that her preference was to do more of this work that indicated care for her feelings than being centred on eating. Kate outlined how therapy sought to enable her to distinguish between the voice of the ED and her own voice and Abbey talked about how externalization enabled her to reclaim both her voice and identity outside of the AN identity.

### Theme 2(b): Life is worth saving—“No one was ever going to give up on me”

Interwoven in participant narratives was a process of ascribing meaning to their parent’s commitment to their recovery.

*EXTRACTS 10*

*Hayley:* *I think I don’t want to forget um, (pause), ah how much care I have seen shine through people in this, like my parents have been supportive the whole time and shows how great they are.*
*Phoenix:* *My parents are really good with supporting me. They keep reassuring me that it's okay. Like, if I'm struggling, they'll be really understanding and they won't force me to do anything that I don't want to do. But they will—they will encourage me.*
*Kate:* *[…] even though I was really angry and did not want to eat anything, mum would still just sit me down and wait*
*Jessica:* *[…] your parents do try to—they try their best to like understand what you’re going through but it’s difficult for them to do that.*
*Maisy:* *[…] how hard it must have been for my parents and how—what a good job they did to persevere and get me to where I am today.*

The participants ascribed a range of meanings to their parents’ support during their treatment, including taking responsibility for their eating in the early phases of FBT. Recollections ranged from parental support and reassurance (Phoenix) and parents’ capacity to be with them when emotionally distressed (Kate). Reflecting back, Jessica connected with her parents’ efforts to “understand” her experience of AN and Maisy with her parents’ perseverance to “get me where I am today”. Furthermore, three participants (Kate, Maisy & Abbey) specifically remembered their parents taking up this role in treatment to avoid them requiring inpatient treatment. These participant experiences highlight the importance of parental support in AN treatments.

For participants who discontinued FBT in the earlier phases, parental support was also noted in their parents’ active collaboration with them to find alternative AN treatments that met their needs and preferences, including treatments that focused on addressing their psychological distress.

*EXTRACTS 11*

*Lydia:* *I had some serious conversations with my parents and I think that I began to sort of get through to them and I think they to some extent also realised that this really wasn’t working, and I needed a different sort of treatment, a different sort of support than what I was receiving.*
*Nora:* *[…] I would say that our relationship [with her mother] now is better than ever and we are able to reflect on the experience and how traumatic it was and how much we both believe the more sick I am is quite harmful to our family and how great it was when we did get individual psychologists.*

Implicit in both these extracts was the significance of these participants being validated in their treatment needs and preferences and specifically for Nora in “how traumatic” AN and its treatment with FBT and hospitalisations were for her and her family. Furthermore, all the participants talked about the significance of their parents standing for them as a person in the face of AN and its treatment.

*EXTRACTS 12*

*Maisy:* *[…] my parents […] were always there telling me that I would get through this and that I was a strong person and—and that no one was ever going to give up on me.*
*Kate:* *Oh, she’s [Mum] just awesome! Like, she is always advocating for my best interest.*
*Charlotte:* *I don’t want to forget that even though, ah, it was very, very traumatising for me, um, that I still have my family and they still stand by me and I stand by them, and even though we went through such a terrible and awful time, um, we still love each other and have each other’s backs during the worst and best periods of our lives. That’s what I—what I wouldn’t want to forget”.*
*Amy:* *Accept the changes that are necessary because ultimately, at the end of the day, people just care about you and they care so much about you that they're going to put you through this. And it's going to be hard, it's going to be really hard, but as soon as you come out the other side and start living your life again and being healthy, the thoughts go away.*

These narratives highlight the significance of the parents standing for these individuals in cultivating a sense of teamwork (Abbey), care (Amy, Phoenix), and that they were not alone (Beth, Jessica). Implicit in all the adolescent narratives was that their parents’ commitment to them and their treatment was hope for their futures and the sense that their lives were worth saving—for example, as depicted by Maisy—“no one was going to give up on me”.

## Discussion

Family-Based Treatment continues to be the frontline treatment for adolescent AN in Australia, although the evidence for it’s  effectiveness in addressing both the physical and psychological symptoms of AN is incomplete. This study sought to understand and give voice to the experiences of adolescents who had either dropped out of FBT or continued to be distressed post-treatment to inform future treatment interventions and research. These young women reported that family support in the context of the treatment was instrumental in saving their lives and contributed to the sense that they were not alone and mattered as a person. Nutritional restoration is non-negotiable [50] in the early stages of any AN intervention to prevent potentially adverse medical outcomes [51]; however, this study has highlighted that delaying interventions to address the individuals’ psychological distress, including in the early stages of treatment, contributed to a loss of voice and/or an exacerbation of their distress. This is consistent with more recent FBT research that has demonstrated the imperative of early treatment focus on not only adolescent weight gain, but also the therapeutic addressing of difficult emotions, criticism of parents towards their child and the alliance between the adolescent/family and the FBT therapist [16].

The current study found the majority of the participants experienced a loss of voice, particularly in the early stages of FBT. This was evident in contexts where their voice was assumed to be the voice of the illness/AN (by parents and/or therapists) thereby externalizing their identities with the AN [24]. Notable in this process, was a disordering of their identities where they were assumed to be incapable of having a valid voice or perspective on their own lives. Furthermore, the neglect of any substantive focus on their psychological distress in the early stages of treatment set the precedent that their internal struggles were of lesser significance. This lack of focus on their psychological distress was cited as a major reason for treatment drop out. Other responses included anger and disengagement from treatment with the sense of themselves as outsiders to their own treatment. This concern has been echoed by Greg Dring [23]:

[…] if the therapist spends the first sixteen sessions of the work discouraging the discussion of feelings, relationship issues and developmental difficulties in a personal way, then it may be very difficult to revive such discussion at a later stage when, in any case, the work is about to be concluded (p. 66).

The participants’ narratives also exemplified how the early treatment focus of FBT on adolescent nutritional restitution inadvertently risked obscuring not only the young persons’ distress but also their family’s systemic distress, which had a recursive effect on their emotional distress. The efforts of parents (as supported by therapists) to encourage their child’s nutritional restoration therefore, at times, unintentionally may have diverted their focus away from holding their child’s less visible, emotional distress. Furthermore, the participants recounted distress and interpersonal strain on their family relationships in the context of parents taking responsibility for their nutritional restoration. It is important to note that some of the parental responses reported by the participants are contraindicated and proscribed in FBT (for example, parental physical restraint). Some of the participants’ experiences highlight possible challenges of parents taking responsibility for their adolescent child’s eating, including whether the ends of adolescent weight gain justify the therapeutic means by which this may be achieved [24], and the imperative of FBT therapists prioritizing the building of a therapeutic alliance [16]. Important in this alliance is a safe context for adolescents and their families to disclose interpersonal difficulties, and that therapists have skills in addressing family conflict, should this arise. Furthermore, in prioritizing the therapeutic alliance, the implementation of the intervention may align more closely with the adolescents preferences for a greater therapeutic focus on addressing intra and inter-personal distress throughout treatment.

Contrary to expectations, the therapeutic alliance, measured by the researcher-rating of the therapeutic alliance (WAI-o), has not been found to be related to AN symptom remission with FBT in previous research [18, 19]. This is a counterintuitive finding when considered with other areas of psychological research where the therapeutic alliance has been shown to predict psychotherapy outcomes more broadly in children and adolescents [52]. The therapeutic alliance is a complex construct and the WAI-o instrument may have been measuring an aspect of the treatment alliance that is not associated with outcomes for FBT with adolescents experiencing  AN. At this stage we do not know. It is an interesting finding that needs further research. However, in keeping with others who have highlighted the importance of listening to a person’s preferences out of respect for their ‘rights and dignity’ and to optimise treatment outcomes [53], this present study indicated that participants valued a strong therapeutic alliance, albeit this may not translate into outcomes that  requires additional research. Therefore, the differences between this and these previous FBT studies may be the methods of assessing and reporting therapeutic alliance and perhaps the construct itself.

Furthermore, the present study findings are consistent with Sibeoni et al. [54] who found that for 15 adolescents who had engaged in both outpatient and inpatient treatment for AN, the therapeutic alliance was facilitated by: the adolescent perception of the treatment team as authentic where they felt heard and understood, themselves and their parents playing an active role in their treatment, time to develop trust with the treatment team with the adolescent-parent relationship as ‘the central element of the therapeutic alliance’. These findings are similar to the experiences of the adolescents in this study, including the importance of a safe therapeutic context to voice their experiences and concerns and to address these, including family difficulties and conflict, should these arise.

The findings of this study are also consistent with Medway and Rhodes’ [36] meta-synthesis into adolescent experiences of family therapy for AN who concluded that psychological interventions for adolescent AN would benefit from scope for interventions that focus more comprehensively on underlying adolescent and family issues. This may go some way to ameliorate the ongoing psychological distress post treatment reported in the current and prior studies [29] and may also prevent the progression on to severe and enduring AN for some [55].

Aspects of the FBT experience were also cited as important in participant recovery journeys. These included parental understanding of their AN experience and standing for them as a person, being treated outside of an inpatient setting, and clarifying a sense of identity outside of the AN identity or “find(ing) out who I was” (Phoenix). These participant experiences highlight the importance of addressing questions of identity in AN treatments [41, 42] and how externalization as an intervention may, in some contexts, facilitate this process. On the other hand, in contexts where the adolescent themselves was excluded from the process of discernment and naming of the AN, externalisation was experienced as invalidating rather than having the intended effect of empowering the person to reclaim their identity from AN [56].

A number of adolescents in this current study reported comorbid depression, anxiety, and/or OCD and all the participants reported experiencing ongoing psychological difficulties irrespective of weight restoration. This is in contrast to recent research that has shown a significant reduction in co-morbid major depressive disorder, generalized anxiety disorder and panic disorder; proposed in those for whom these conditions are likely to be secondary to malnutrition [57]. Notably, a third of participants retrospectively reported escalating suicide risk during the treatment intervention. This is of concern, given the findings that half of all deaths in AN result from suicide [3, 4]. Consistent with previous research [36] and an Australian Broadcasting Commission medical report in 2017 (https://www.abc.net.au/news/2017-05-04/australian-health-system-failing-patients-with-eating-disorders/8485300), the participants in this study argued for more holistic approaches to AN treatments and that, in their experiences, there was limited scope for FBT to be tailored to their individual needs and preferences, thereby contributing to the decision for some of the participants to cease treatment prematurely and/or continuance of ongoing psychological distress post-treatment.

The importance of addressing an adolescents’ psychological distress has been increasingly recognized over the past decade with a number of FBT treatment augmentations being proposed and researched. These have included multi-family therapy [58, 59], parent-to-parent consultations [60], separated-family therapy [61], addition of psychological interventions such as CBT [62] and Dialectical Behavior Therapy (DBT) [63] and therapist guided internet chat rooms [64]. These augmentations have tended to focus on changing the context of treatment, providing additional or novel means of support in addition to treatment as usual, particularly for parents [65], rather than augmentation to the structure and/or transformation of content of the treatment intervention itself. The structural augmentation to separate parent and adolescent sessions, particularly in phase one of FBT, has found similar weight gain outcomes, irrespective of whether the adolescent is involved in the early phases, with increased retention and remission rates for some adolescents [14]. Furthermore, the majority of augmentations involve phase one only and are reflective of the reluctance to change or ‘tamper’ with the manualized intervention, despite the call for more ‘potent augmentations’ to improve outcomes [60]. There continues, however, to be a paucity of researched interventions that have scope to transform the landscape of treatment options for anorexia nervosa.

### Clinical questions

This research study has explored the treatment preferences of 14 adolescents who either dropped out or continued with psychological distress post FBT and the following questions need to be interpreted in light of this. These participants’ experiences prompt consideration of the following questions by clinicians and researchers in relation to future treatment interventions for adolescent AN:

1. What is going on for the adolescent, including their emotional life [23]?
2. How might the adolescent’s’ voice [23] and personal agency be prioritized as they undertake the challenging task of nutritional restoration in the early stages of treatment within a treatment non-negotiable framework [50]?;
3. What is going on for the family and how might problematic family dynamics, including family conflict, be addressed?
4. At what point and how do therapists assess whether the allocation of responsibility to the parents is working and when this intervention might be contraindicated for an individual family?;
5. How might interventions more comprehensively address complex identity negotiations for adolescents and their family members? [45]; and
6. To what extent might interventions be flexibly tailored to the adolescent and family needs and preferences within a treatment non-negotiable framework [50] that prioritizes and supports the adolescent’s physical and psychological safety?

Furthermore, future treatments might consider what is already becoming increasingly recognized, that family treatments for adolescent AN need to consider behavioural, psychological symptomology and family functioning in parallel and to map treatment effectiveness to these outcome measures [26].

### Study strengths and limitations

The current study recruited a population of whom the majority had either dropped out and/or continued with ongoing psychological distress post treatment with FBT. Qualitative papers are, by design, in-depth investigations of small samples. However, it is usual to continue to interview or seek data until thematic saturation is reached. As the wording of the advertisement indicated inclusion of those who had discontinued FBT, this may have inadvertently excluded participants who continued to experience psychological distress post FBT suggesting that further research on this group is indicated. With this targeted recruitment of participants there exists a risk of negative bias in participants’ recollection of their FBT treatment experiences in the event that participants subsequently engaged in more positively experienced treatments. On the other hand, this study goes some way in mitigating against the risk of positive bias in participant recollection of their treatment experiences that may arise in the context of a positive outcome and recovery. Another strength of the study was two author extraction of data to reduce the risk of bias in interpretation of the transcript data. Furthermore, the scope of this study is in the development of a better understanding of why FBT does work for all and why.

Negative experiences in a treatment intervention are important to understanding, however, empirical study of methods of change are required to suggest modifications to treatment that may lead to improved outcomes. It is not possible to know whether, if these adolescents’ treatment preferences had been met at the time of treatment with FBT, treatment outcomes would have been improved. Also unknown were the skills and training of the FBT therapists’ who delivered the individual interventions, including their capacity to build a therapeutic alliance and address the adolescent and/or family’s distress.

What is known, is that the findings would suggest further research is needed to test, and find ways to test, the clinical questions that have been raised in this paper in regard to improving both treatment engagement and outcomes for adolescent AN. Future research may also seek to conduct similar research to explore the experiences and perspectives from a more diverse group of adolescents with a lived experience of adolescent AN. Further research would benefit from qualitative data triangulation (from multiple sources including adolescent, parent and clinician experiences of FBT) for completeness, convergence and dissonance of the key themes identified in this paper.

## Concluding remarks

This current study highlights the complexity that is involved in the treatment of adolescent AN and consideration of systemic family issues, adolescent psychological distress and identity formation in family-based treatments. Many adolescents experience physical and psychological symptom improvement with FBT. Nevertheless, the need for greater focus on addressing psychological distress, in all phases of treatment, was identified by all the adolescents in this study as putative changes that would improve experiences and outcomes in AN treatments. Further research is needed to extend the current findings, including the tailoring of treatment to the adolescent and family needs and preferences [66] and addressing more comprehensively questions of identity, and how they may be applied in the development and evaluation of transformative AN treatment interventions.

## Data Availability

The datasets used and/or analysed during the current study are available from the corresponding author upon reasonable request.

## Appendix A

### Research study advertisement

This study is interested in hearing the voices of those who have experienced Family-based therapy for anorexia nervosa and decided for whatever reason to not continue in this treatment. We are interested in hearing what it was like for you and your family to experience Family-Based Therapy for anorexia nervosa as we believe that your experiences are important to consider when developing new ways of treating anorexia.

## Appendix B

### Interview schedule

A selection of questions will be used with each participant and questions will scaffold between.

- Experience (e.g. Can you tell me about …?)
- Meaning (What does … mean to you?)
- Identity (e.g. How does … have you seeing yourself as a person, what does this say to you about what matters to you as a parent/what is important to you/what you value?)
- Positioning on experience/identity conclusion (e.g. is this OK for you or not? Why?)

**(A) Interview will be augmented with a timeline for narrative/discourse**

1. Can you tell me your story of anorexia?
2. When did anorexia start to have an impact on your life? What was this like for you as a person? And as a child in your family? How did this have you seeing yourself as a person? Was this helpful or not? Why?
3. As you look back on your experiences of anorexia*, which events stand out in your mind?
4. As you look even further back before anorexia*, which events stand out in your mind?
5. Can you tell me a bit about your relationship with anorexia now? How does this affect your life? Relationships? How you see yourself as a person/child?
6. Do you consider your relationship with anorexia* to be problematic for you now? If so, how? If not, why?
7. What does having experienced anorexia mean to you as a person?
8. Has your life changed with your experience of anorexia*? What have you got in touch with yourselves as a person/daughter/son?

**(B) Experiences of Family-Based treatment**

1. How did you come to be involved in Family-Based treatment (FBT)?
2. Can you tell me about your experiences of FBT?
3. What was most helpful about FBT? Why?
4. What was least helpful about FBT? Why?
5. Did you complete FBT? Why or why not?
6. Did FBT assist you to shift your relationship with anorexia?
7. Overall, what stands out for you if you reflect on what impacts FBT had on you? Was this helpful or not? Why?
8. How has participating in the FBT affected how you see yourself as a person? Is this helpful or not? Why?

**Then ask questions from (C)** **OR** **(D)**

**(C) If the adolescent/family completed FBT**

1. When did you finish FBT?
2. What has your relationship with anorexia been like since you completed FBT? How has this been for you as a person and as a family since discontinuing FBT?

OR

**(D) If the adolescent/family discontinued FBT**

1. At what phase of FBT did you discontinue treatment?
2. What has your relationship with anorexia been like since then? How has this been for you as a person and as a family since discontinuing FBT?
3. Can you tell us about the circumstances/your experiences that led you to no longer continue with FBT?
4. Looking back how do you make sense of why you chose not to continue with FBT?
5. Are you settled with the decision or not? Why?
6. Did you have any fears/concerns about what might happen if you were to continue or discontinue with FBT?
7. What were you hoping for as you made the decision to cease FBT?

**(E) Questions to all participants regardless of whether completed or discontinued FBT**

1. What do you want to not forget about yourself from the FBT?
2. What might be important for another person/family to know as they commence FBT?
3. What advice might you give to a person or family who is about to start FBT?
4. What would you like more of in future treatments for anorexia? What would you like less of in future treatments for anorexia nervosa? What does this say to you about what matters for you/your child/ your family?
5. Do you have hopes for future treatment/therapy in terms of the sort of work you would like to engage in? What does this say to you about what you value as a person?
6. Has our conversation today been helpful or unhelpful or both? Why?
7. What has stood out for you from our conversation today?
8. What might be important for us not to forget as we analyse the data from this interview?

## Appendix C

### Researcher positioning statements

**Janet Conti**: I am a Clinical Psychologist, Dietitian and academic in Clinical Psychology. My research and clinical work seeks to prioritise the voice of the experiencing person to inform the development of a greater number of effective treatment inteventions for AN that are tailored to the needs and preferences of the experiencing person and their family. This research is one arm of a larger research project that is aimed to give voice to adolescents, parents and the clinicians who treat them about their experience of FBT as the first line of treatment for adolescent AN in Australia.

**Caroline Joyce**: I am an academic in medicine with a background in Health Psychology. My research explores the impact of psychosocial factors on people adjusting to illness and chronic diseases. I am particularly interested in how psychological distress impacts treatment adherence and recovery from illness to better understand more effective treatments.

**Simone Natoli**: I am a Clinical Psychologist currently working in private practice with a number of clients diagnosed with eating disorders. I have an interest in understanding the best treatments available and how to maximise treatment outcomes. Understanding the factors contributing to poor treatment outcomes is an important part of this approach.

**Kelsey Skeoch**: I am a Clinical Psychologist currently working in a Child and Adolescent Mental Health Service for the National Health Service (NHS) in the United Kingdom. I have a keen interest in the mental health of children and young people, and in particular how the systems in which young people live and access, can both promote and hinder treatmemt outcomes.

**Phillipa Hay**: I am an academic Psychiatrist with long-standing clincial and research experience in the treatment of people with anorexia nervosa. I am very interested to explore and understand better how to improve treatments, reduce distress during treatment, and in particular to better understand why some people have poor outcomes.

## Appendix D

### Participant member check feedback (participant pseudonyms)

**Lydia:** I have read over the paper and am happy with the way my testimony has been used. Thank you for keeping it authentic. I eagerly await publication of the final paper. I feel it will prove instructive for my parents, going forward.

**Harley**: Addition to extract 2: “The FBT Program in my opinion has a narrow focus on food and weight restoration, rather than overall psychological wellbeing. For me it was harder than what it should have been because FBT unmasked other psychological issues which the anorexia had been used to deal with. The ‘manualised’ program approach failed to accommodate individual needs”.

**Amy:** lt looks great though, very interesting report.

**Charlotte:** I am happy with the paper and would be most grateful to receive a copy of the paper if published. Thank you very much for including me in the study.

(Note: Some participants were unable to be contacted).

## Abbreviations

AN: Anorexia Nervosa
ED: Eating Disorder
DBT: Dialectical Behavior Therapy
FBT: Family-Based Treatment
OCD: Obessive Compulsive Disorder
